# Completeness of intervention description in invasive cardiology trials: an observational study of *ClinicalTrials.gov* registry and corresponding publications

**DOI:** 10.3389/fmed.2023.1276847

**Published:** 2023-10-10

**Authors:** Viktoria Lišnić, Hishaam Ashraf, Marin Viđak, Ana Marušić

**Affiliations:** ^1^Department of Cardiology, University Hospital of Split, Split, Croatia; ^2^Wirral University Teaching Hospital, Wirral, United Kingdom; ^3^Department of Research in Biomedicine and Health, Center for Evidence-Based Medicine, University of Split School of Medicine, Split, Croatia; ^4^Department of Cardiology, Dubrava University Hospital, Zagreb, Croatia

**Keywords:** cardiology, TIDieR checklist, *ClinicalTrials.gov*, quality of reporting, non-pharmaceutic interventions

## Abstract

**Introduction:**

Non-pharmacological invasive interventions in cardiology are complex and often inadequately reported. Template for Intervention Description and Replication (TIDieR) checklist and guide were developed to aid reporting and assessment of non-pharmacological interventions. The aim of our study was to assess the completeness of describing invasive cardiology interventions in clinical trials at the level of trial registration and corresponding journal article publication.

**Methodology:**

We searched for clinical trials in invasive cardiology registered in *Clinicaltrials.gov* and corresponding journal publications. We used the 10-item TIDieR checklist for registries and 12-item checklist for journal publications.

**Results:**

Out of 7,017 registry items retrieved by our search, 301 items were included in the analysis. The search for corresponding published articles yielded 192 journal publications. The majority of trials were funded by the industry and were medical device trials. The median number of reported TIDieR items was 4.5 (95% CI 4.49–4.51) out of 10, and while the corresponding journal articles reported 6.5 (95% CI 6.0–6.5) out of 12 TIDieR items.

**Conclusion:**

Registration and reporting of invasive cardiology trials is often incomplete and adequate detailed description of the interventions is not provided. TIDieR checklist is an important tool which should be used to ensure rigorous reporting of non-pharmacological interventions in cardiology.

## 1. Introduction

Non-pharmacological invasive interventions in medicine, such as those in invasive cardiology procedures, are often complex. Their development, adoption, and assessment of their efficacy are challenged by different factors (1). These also include reporting of trials of such interventions, so that the reports ensure adequate presentation of randomized controlled trials (RCT) as minimally biased, highly reliable sources of evidence (2, 3).

The development and evaluation of non-pharmacological interventions have phases that importantly differ from those for pharmacological interventions. In pharmacological research, innovation is tightly controlled in a series of processes, and the majority of those are conducted before the drug is approved for broad human use, that is, its design and adoption are separated (4). For non-pharmacological interventions, innovation of a procedure continues as it is adopted into practice, in stages, as described in the Idea, Development, Exploration, Assessment and Long-term study (IDEAL) Framework and Recommendations in 2009 (5). Any opportunity for formal assessment will thus need to be sought during the early period of adoption of a new surgical operation (4).

Traditionally, those who perform invasive interventions have selected and assessed the outcomes themselves, reporting on short-term clinical outcomes of technical success and harm (6). The reporting of those outcomes is not standardized and often not reproducible, hindering methodological assessment, comparison of interventions and translation to clinical practice (5). Without a complete published description, clinicians and patients cannot reliably implement interventions that are shown to be useful, and other researchers cannot replicate or build on research findings. The quality of description of interventions in publications, however, is remarkably poor (7). Despite calls for surgical and other invasive interventions research to be more rigorous, the overall frequency of RCTs for invasive procedures has been consistently low since the 1970s (8). Since then, reports of non-pharmacological intervention studies still suffer from small sample sizes and reporting bias, with suboptimal registration and lacking the assessment of the quality of intervention (9).

To improve the completeness of reporting of interventions, and non-pharmacological interventions in particular, Template for Intervention Description and Replication (TIDieR) checklist and guide were developed by an international group of experts as an extension of the CONSORT 2010 and the SPIRIT 2013 statement. TIDieR checklist ensures that the most important information is provided about the intervention tested in a trial (7), and is relevant for both the information that has to be registered in a trial registry and in journal publication. Despite the availability of the TIDieR checklist for almost 10 years, the adherence to TIDieR checklist in cardiology interventions is still poor (9) and scientific journals do not require nor endorse the use of this checklist (10).

To our knowledge, no assessment of the completeness of reporting of registry items and publications for non-pharmacological interventions has been conducted for procedures in invasive cardiology. This study aimed the assess the completeness of reporting interventions in clinical trials in invasive cardiology, both at the level of trial registration and corresponding journal publication.

## 2. Methods

### 2.1. Study design and setting

This was an observation, cross-sectional study of invasive cardiology clinical trials registered at *Clinicaltrials.gov* trial registry, as well as matching publications. We defined invasive procedure in cardiology as a complex intervention with deliberate access to the body via an incision or percutaneous puncture, with instrumentation used in addition to the puncture needle (11). We used STROBE (Strengthening the reporting of observational studies in epidemiology) checklist for reporting results (12).

### 2.2. Sample and inclusion criteria

We developed a search strategy to identify clinical trials in invasive cardiology by searching for completed clinical trials with results, using the following search string: invasive AND (cardiology OR artery OR bypass OR cardiac OR cardiovascular OR coronary OR heart OR myocardial OR stent OR vessel). To be included in the study, registered trials had to: (1) be closed and completed at the time of our search according to Overall Recruitment Status in the registry, (2) with reported study results in *Clinicaltrials.gov* and (3) had non-pharmacological invasive cardiology intervention noted in one or more following registration fields within the Descriptive Information section of the *ClinicalTrials.gov*. Studies of unknown status, observational or studies still enrolling participants were excluded. *Clinicaltrials.gov* was searched on 25 September 2019 (using the classic version of the website)^1^ and followed up to allow at least 2 years for the publication of trial results in a journal. Trials with registered results were chosen for the study sample because they had higher chance to have a journal publication.

Two authors (VL, HA) independently screened the retrieved items. There were no disagreements. After screening and identifying invasive cardiology trials registered in *Clinicaltrials.gov*, two authors (VL, MV) independently searched corresponding publications on 26th May 2023, which were identified by screening the following sources: (1) the Publications subheading under the *ClinicalTrials.gov* Descriptive Information heading (displayed under Tabular view), (2) PubMed/MEDLINE, and (3) Scopus. The manual search used (1) trial unique identification number, and (2) combination of search terms for each trial: intervention name, condition, study phase, and all names under “investigators” field in *Clinicaltrials.gov*. If there were more than one corresponding journal publication available, we analyzed the first publication, which presented the results related to the primary outcome.

### 2.3. Data extraction

MV developed a data charting form, which was reviewed by VL and AM. VL and HA independently extracted data for the following items: NCT number, title, acronym (where available), study type, status, study results, conditions, intervention (where applicable), type of intervention (as provided in the *Clinicaltrials.gov*) comparator (where applicable), outcome measures, funders, sponsors, locations, participant characteristics (gender, age), study phase, enrolment status, participant size, study design, availability of study documents, study start dates, primary completion dates, availability of the results and results dates.

To evaluate the completeness of reporting invasive cardiology interventions, we adapted the 12-item TIDieR checklist. Checklist used in our study included the items from the TIDieR checklist: Item (1) Brief name; Item (2) Why; Item (3) Materials (we separately checked if the manufacturer of the device (3a), type of device used (3b) and specifics of the device (3c) were provided); Item (4) Procedures (we separately checked if the place of entry of the device (4a), preparation for the procedure (4b) and sequence of procedure steps in intervention (4c) were reported); Item (5) Provider (we separately analyzed if the background and job roles of the providers (5a) as well as prior expertise, training and education and competence assessment (5b) was reported); Item (8) When and How much included expected duration of the intervention and if applicable, a number of sessions or intervals of the intervention; Item (9) Tailoring, how the intervention was adapted for individuals in the study; Item (10) Modifications, how the interventions was modified during the study (including changes in the intervention, not the outcomes measured); Item (11) and (12) how well was the intervention adhered to, either planned or actual. Items 10 (Modifications) and 12 (Actual adherence to the intervention procedure) were not analyzed at the level of the registry, as instructed by the TIDieR guide (6). For published articles, a full TIDieR checklist was used.

Completeness of TIDieR checklist item reporting in the *Clinicaltrials.gov* registry was assessed by two researchers (VL, HA) independently, and a third author (MV) was consulted to resolve discrepancies. HA is a medical doctor, VL is a cardiology resident and a PhD student and MV is a cardiology resident and a researcher with special interest in evidence based medicine and research methodology. The kappa coefficient between the two assessors ranged from 0.72 to 0.94 for individual TIDieR items. Completeness of reporting TIDieR checklist items in the corresponding publications was assessed by two researchers (VL, MV) independently, with no disagreements between the researchers.

### 2.4. Data analysis

The overall completeness of reporting of interventions was measured as the median of TIDieR checklist items reported. One point was given for complete compliance and no points were given for noncompliance; for checklist items (3) Materials, (4) Procedures, (5) Providers, one point was given if all details were reported and 0.5 points were given for partial reporting if at least half of the subitems were reported, following the methodology from study conducted by Palmer et al. (11). Items 9 (Tailoring), 10 (Modifications), 11 (Planned adherence to the intervention procedure) and 12 (Actual adherence to the intervention procedure) were considered noncompliant unless they were not reported unnecessary or not required by the study. Inadequate reporting for other checklist items were considered non-reporting. Statistical analysis was conducted using MedCalc Statistical Software version 14.8.1 (MedCalc Software, Ostend, Belgium).^2^ Descriptive statistics were used to present the collected data. Categorical variables were presented as frequencies, absolute values or percentages and continuous variables as mean or median values with 95% confidence intervals, depending on the distribution of the data.

## 3. Results

**Figure 1:**
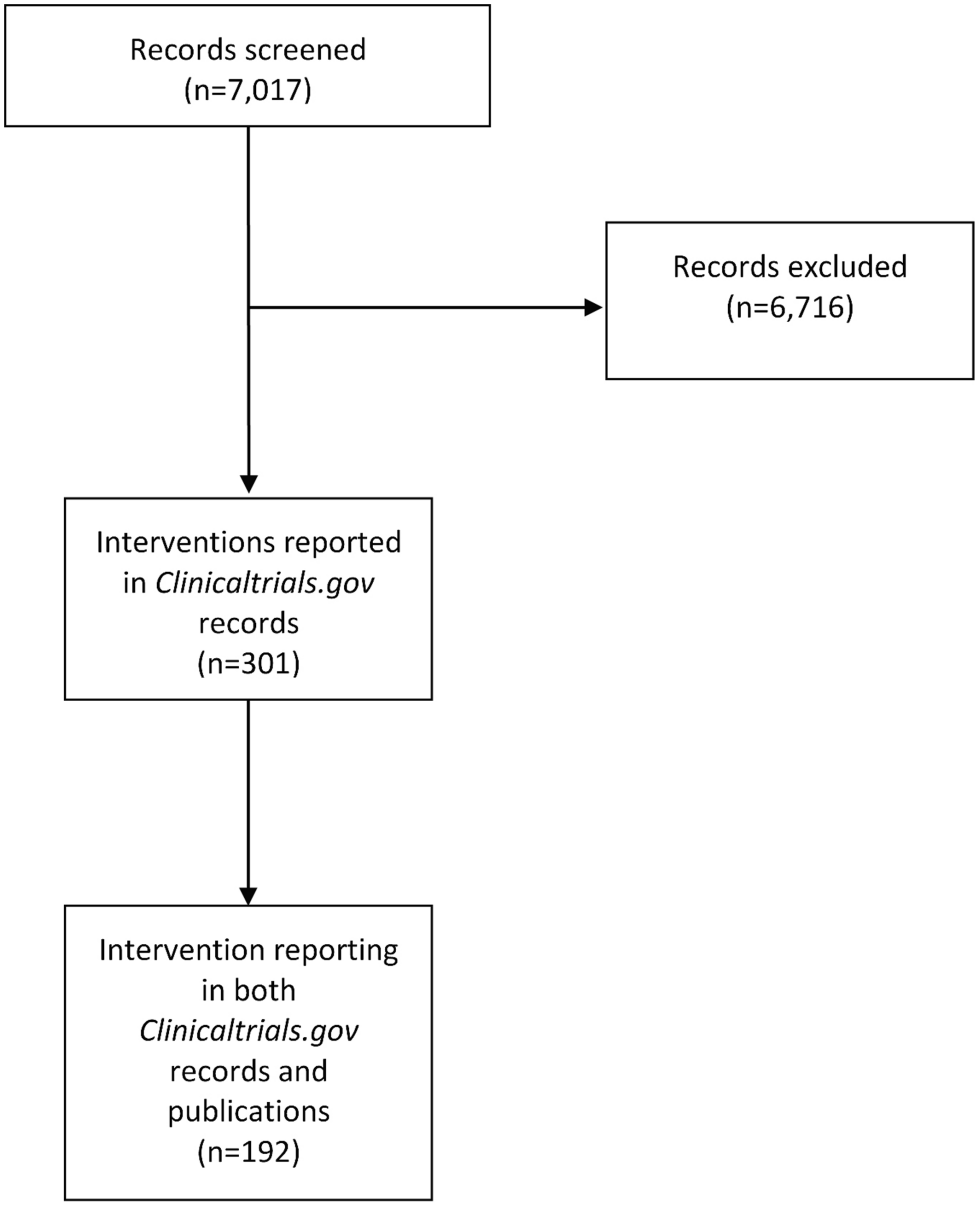
Study flow diagram for the selection of eligible interventional trials in invasive cardiology.

The search of *Clinicaltrials.gov* retrieved 7,017 results (Figure 1). After screening of titles and descriptions of retrieved items, 6,716 were excluded (659 were not reporting invasive procedures, 107 were not interventions in cardiology, 94 were dealing with peripheral artery disease and 36 were dealing with cardiac surgery), leaving 301 items to be included in the analysis. The search for corresponding published articles yielded 192 journal publications.

### 3.1. General characteristics of registered trials

Characteristics of reported trials are presented in Table 1. The majority of analyzed trials described the interventions in treating arrythmias and conduction disorders (39.8%), followed by coronary artery disease (32.9%), and heart failure (14.3%). The primary purpose for the majority of studies was reported as treatment (80.4%), and the majority of studies were conducted in North America (46.5%). Study types were often open (78.4%) and non-randomized trials (61.4%). The majority of trials were funded by industry (83.4%) and were categorized as device trials (86.7%). There were no studies with more than one registered sponsor in our sample. The majority of interventions were testing devices, and the most registered interventions dealt with arrythmias and conduction disorders, with the most common being arrythmia ablations. The list of most common interventions registered for different conditions is available in the Supplementary Table S1.

**Table 1:** Characteristics of the registered trials on invasive cardiology interventions with closed and completed overall recruitment status and with registered results in *Clinicaltrials.gov* (*n* = 301).*

Characteristics	Number (%)
Research area	
Arrythmias and conduction disorders	120 (39.8)
Coronary artery disease	99 (32.9)
Heart failure	43 (14.3)
Valvular disease	23 (7.6)
Other	9 (2.9)
Congenital heart defects	7 (2.3)
Primary purpose	
Treatment	242 (80.4)
Diagnostic	20 (6.6)
Prevention	14 (4.6)
Other	14 (4.6)
Supportive care	9 (2.9)
Basic science	2 (0.6)
Region	
North America	140 (46.5)
Europe	79 (26.2)
Other	50 (16.6)
Asia	24 (7.9)
South America	8 (2.6)
Study design	
Single group	179 (59.5)
Parallel	115 (38.2)
Cross over	6 (2.0)
Factorial	1 (0.3)
Sample size	
Estimated enrollment (C, 95% CI)	158 (120–198)
Randomization	
Non-randomized	185 (61.4)
Randomized	116 (38.5)
Masking	
None	236 (78.4)
Single	47 (15.6)
Double	15 (4.9)
Quadruple	2 (0.6)
Triple	1 (0.3)
Research phases	
Not reported	205 (68.1)
Phase 4	44 (14.6)
Phase 3	35 (11.6)
Phase 2	13 (4.3)
Phase 1&2	3 (1.0)
Phase 2&3	1 (0.3)
Early phase	0
Phase 1	0
Sponsor	
Industry	251 (83.4)
Academic	49 (16.3)
NIH	1 (0.3)
Type of intervention	
Device	261 (86.7)
Procedure	34 (11.3)
Other	6 (2.0)

*Search strategy: invasive AND (cardiology OR artery OR bypass OR cardiac OR cardiovascular OR coronary OR heart OR myocardial OR stent OR vessel).

### 3.2. Completeness of intervention descriptions in *ClinicalTrials.gov*

The trial protocols registered in *ClinicalTrials.gov* reported a median of only 4.5 (95% CI 4.49–4.51) out of 10 analyzed TIDieR items (ttems 10 and 12 were not analyzed at this step) (Table 2). TIDieR item 1 (Brief name) was present in all 301 trials reviewed. Reporting was also complete (>90%) for TIDieR item 2 (Why), item 3a (Manufacturer of the device) and item 7 (Location). However, the specifics of the device (TIDieR item 3c) were not reported in more than two-thirds of the registered interventions.

**Table 2:** The completeness of invasive cardiology intervention descriptions in *ClinicalTrials.gov*^1^ (*n* = 301).

TIDieR item	Present	Not present	Not applicable
1 – Brief name	301 (100)	-	
2 – Why (background info)	279 (92.7)	22 (7.3)	
3 – What (materials)^2^			
3a – Manufacturer	254 (84.4)	47 (15.6)	
3b – Type	282 (93.7)	19 (6.3)	
3c – Specifics	208 (69.1)	93 (30.9)	
4 – What (procedures)^3^			
4a – Place of entry of the device/intervention	68 (22.6)	227 (75.4)	6 (2.0)^4^
4b – Preparation	12 (4)	289 (96)	
4c – Sequence of procedure steps in intervention	32 (10.6)	269 (89.4)	
5 – Intervention provider^5^			
5a – Provider: background	24 (7)	277 (93)	
5b – Provider: additional training, competence assessment	7 (2.4)	294 (97.6)	
6 – How: Delivery mode	301 (100)	-	
7 – Where	292 (3)	292 (97)	
8 – When and how much		301 (100)	
9 – Tailoring	2 (0.7)	299 (99.3)	
11 – How well (planned): Intervention adherence	2 (0.7)	299 (99.3)	

^1^TIDiER Items 10 and 12 were not analyzed as they are not relevant for registered information (Hoffmann 2014). ^2^TIDieR Item (3) What (materials) includes information on the materials used in the interventions: manufacturer (3a), type of device used in the intervention (3b), and specifics of the device (3c). ^3^TIDieR Item (4) What (procedures) describes each of the procedures in the intervention, including the place of entry for the device used in the invasive cardiology (4a), preparation for the intervention (4b) and the detailed sequence of procedure steps in the interventions (4c). ^4^Not applicable: In 6 trials the study involved the intervention to reprogramme a device implanted previously. ^5^TIDieR item (5) Intervention provider describes who was involved in providing the intervention, by providing information on the disciplinary background and job roles and skills (5a) and additional training and prior competence assessment (5b).

Place of entry of the device (TIDieR item 4a) was not provided in 75.4% of registered trials, information on preparation (TIDieR item 4b) was not present in 96% of trials, and the sequence of procedure (TIDieR item 4c) was not present in 89.4% of trials. Details on providers of the intervention and their previous education or training were not reported for the majority of trials (93, and 97.6%, respectively). TIDieR item 8 (When and how much) was not reported for any of the registered trials. TIDieR item 9 (Tailoring) and item 11 (Planned adherence to the intervention procedure) were not reported for the majority of trials (99.3%).

### 3.3. Completeness of intervention descriptions in corresponding journal articles

Of 301 trials posted to *Clinicaltrials.gov*, 191 had the results published in journal articles (Table 3). A median of 6.5 (95% CI 6.0–6.5). TIDieR items were reported in the corresponding journal articles. TIDieR items 1 (Brief name), 2 (Background info), 3 (Manufacturer and specifics of the device), and 7 (Location) were reported most often. TIDieR 5 (Intervention provider) was not provided in more than 60% of publications. TIDieR items 8 (Total duration of the intervention), 9 (Tailoring), 10 (Modifications) and 12 (Actual changes described in adherence to the intervention procedure) were not reported in more than 90% of publications.

**Table 3:** The completeness of invasive cardiology intervention descriptions in published articles (*n* = 192).

TIDieR item	Present	Not present	Not applicable^3^
1 – Brief name	192 (100.0)	-	
2 – Why (background info)	192 (100.0)	-	
3 – What (materials)^1^			
3a – Manufacturer	163 (84.9)	29 (15.1)	
3b – Type	178 (92.7)	14 (7.3)	
3c – Specifics	150 (78.1)	42 (21.9)	
4 – What (procedures)^2^			
4a – Place of entry of the device/intervention	123 (64.1)	67 (34.9)	3 (1.0)
4b – Preparation	132 (69.3)	59 (30.7)	
4c – Sequence of procedure steps in intervention	119 (62.0)	73 (38.0)	
5 – Intervention provider^4^			
5a – Provider: background	65 (34.9)	127 (66.1)	
5b – Provider: additional training, competence assessment	27 (14.1)	165 (85.9)	
6 – How: Delivery mode	192 (100.0)	-	
7 – Where	154 (80.2)	38 (19.8)	
8 – When and how much (total duration)	-	192 (100.0)	
9 – Tailoring	6 (3.1)	186 (96.9)	
10 – Modifications during the course of study		192 (100)	
11 – How well (planned): Intervention adherence	115 (59.9)	77 (40.1)	
12 – How well (actual): Intervention adherence	45 (23.4)	147 (76.6)	

^1^TIDieR Item (3) What (materials) includes information on the materials used in the interventions: manufacturer (3a), type of device used in the intervention (3b), and specifics of the device (3c). ^2^TIDieR Item (4) What (procedures) describes each of the procedures in the intervention, including the place of entry for the device used in the invasive cardiology (4a), preparation for the intervention (4b) and the detailed sequence of procedure steps in the interventions (4c). ^3^Not applicable: In 6 trials the study involved the intervention to reprogramme a device implanted previously. ^4^TIDieR item (5) Intervention provider describes who was involved in providing the intervention, by providing information on the disciplinary background and job roles and skills (5a) and additional training and prior competence assessment (5b).

### 3.4. Comparison of intervention descriptions in *ClinicalTrials.gov* and corresponding journal articles

A comparison of intervention descriptions for trials registered in *ClinicalTrials.gov* and in corresponding journal articles is presented in Table 4. TIDieR items 1 (Brief name), 2 (Background information) and 7 (Location) were most often present in both *Clinicaltrials.gov* and in matching publications. The manufacturer of the device (TIDieR 3) was described in both registry and publication for the majority of trials, while the specifics of the device (TIDieR 3), preparation for the procedure and sequence of procedure steps in the intervention (TIDieR 4) were more often reported in published articles. TIDieR item 11 (Planned adherence to the intervention procedure) was reported in both registry and published articles. TIDieR item 5 (Intervention provider), item 8 (When and how much) and item 9 (Tailoring) were mostly unreported both in the registry and the matching publication.

**Table 4:** Comparison of invasive cardiology intervention descriptions in *ClinicalTrials.gov* corresponding journal articles (*n* = 192).

TIDieR	Present in both	Article only	CT.gov only	Not present in article or registry	Not applicable^3^
1 – Brief name	192 (100.0)	-	-	-	-
2 – Why (background info)	179 (93.2)	13 (6.8)	-	-	-
3 – What (materials)^1^					
3a – Manufacturer	143 (74.5)	20 (10.4)	16 (8.3)	13 (6.8)	-
3b – Type	169 (88)	8 (4.2)	8 (4.2)	7 (3.6)	-
3c – Specifics	57 (29.7)	93 (48.4)	8 (4.2)	34 (17.7)	-
4 – What (procedures)^2^					
4a – Place of entry of the device/intervention	23 (12.0)	44 (22.9)	22 (11.5)	100 (52.1)	3 (1.6)
4b – Preparation	3 (1.6)	129 (67.2)	3 (1.6)	57 (29.7)	-
4c – Sequence of procedure steps in intervention	13 (6.8)	106 (55.2)	9 (4.7)	64 (33.3)	-
5 – Intervention provider^4^					
5a – Provider: background	2 (1.0)	63 (32.8)	11 (5.7)	116 (60.4)	-
5b – Provider: additional training, competence assessment	1 (0.5)	26 (13.5)	3 (1.6)	162 (84.4)	-
6 – How: Delivery mode	192 (100.0)	-	-	-	-
7 – Where	150 (78.1)	4 (2.1)	38 (19.8)	-	-
8 – When and how much (total duration)	-	-	-	192 (100.0)	-
9 – Tailoring	-	6 (3.1)	1 (0.5)	185 (96.4)	-
11 – How well (planned): Intervention adherence	114 (59.4)	-	1 (0.5)	77 (40.1)	-

^1^TIDieR Item (3) What (materials) includes information on the materials used in the interventions: manufacturer (3a), type of device used in the intervention (3b), and specifics of the device (3c). ^2^TIDieR Item (4) What (procedures) describes each of the procedures in the intervention, including the place of entry for the device used in the invasive cardiology (4a), preparation for the intervention (4b) and the detailed sequence of procedure steps in the interventions (4c). ^3^Not applicable: In 6 trials the study involved the intervention to reprogramme a device implanted previously. ^4^TIDieR item (5) Intervention provider describes who was involved in providing the intervention, by providing information the disciplinary background and job roles and skills (5a) and additional training and prior competence assessment (5b).

## 4. Discussion

Our study showed that registration and reporting of invasive cardiology trials are often incomplete, with adequate detailed description of the interventions not provided. Whereas the number of items describing trial intervention increased from the registration to the published data, the information in the published articles often differed from those in matching registry records. This means that it is rather difficult to directly translate new interventions and procedures into clinical cardiology practice.

TIDieR checklist enables precise and structured reporting of complex interventions by facilitating a clear and detailed description of the intervention, regardless of the study design (7, 13). TIDieR checklist can also be used as a quality rating scale (14) and help reviewers during the peer review process (7). TIDieR checklist was originally devised as an extension to the CONSORT reporting guideline, where only one of the 25-item checklists was dedicated to intervention description (15). Low endorsement of the TIDieR checklist is still prevalent, and in a recent call to action, Ryan et al. asked journal editors to update their submission guidelines by making a separate TIDieR checklist mandatory for interventional trials (10). TIDieR checklist enables the implementation and replication of research findings and facilitates transparent reporting of results, supplementing good clinical practice and responsible conduct of research.

TIDieR checklist has previously been used to assess interventions in rehabilitation medicine (16, 17), surgery (18), educational (19) and public health interventions (10), as well as a tool to assess interventions used in systematic reviews (20, 21). In cardiology, a single study looked at adherence to the TIDieR checklist in cardiology journals, which included only higher-impact journals (9). They found higher adherence to the TIDieR checklist (median 8.6 items) than in our study (median 6.5 out of 12 items for published articles). Such differences could be explained by a selective search for high-quality journals which are more likely to adapt and endorse the usage of reporting guidelines (22) and by a higher percentage of pharmacological (drug) interventions in the analyzed sample, which are often better reported (23).

Reporting of non-pharmacological interventions is particularly challenging and the quality of reporting of such trials is lower in comparison to research on pharmacological interventions (24). Quality of reporting of complex interventions is not improving, despite the endorsement of both CONSORT and TIDieR checklists (25). A potential barrier to detailed reporting of interventions could be the word limit for manuscripts in journals. Analysis of RCTs on non-pharmacological interventions in physical therapy and stroke interventions using the TIDieR checklist yielded results similar to our own (16, 17). A previous analysis of reporting of surgical interventions showed poor adherence to the TIDieR checklist (26). A systematic review of non-pharmacological interventions in Crohn’s disease showed that no studies had coverage of all domains of TIDieR (18). In a recently published study on nonsurgical periodontal therapy, adherence to the TIDieR checklist was also low, with discrepancies between registries and published articles (27).

TIDieR items which were most often reported were items 1 (Brief name), 2 (Why) and 7 (Where), both in the clinical trial registry as well as in matching publications. TIDieR item 4 (Procedures), which was not reported in more than two third of the trials, is essential in invasive cardiology. For example, transcatheter aortic valve replacement (TAVR) is usually done using the trans-femoral approach and for a selected population of patients, alternative routes (such as transaortic or transapical) can be used (28) as the choice of access in TAVR seems to be independently associated with an impact on prognosis, transparent reporting of the place of entry is critical.

Previous training and experience of those conducting the intervention were also underreported (item 5). Invasive cardiologists are expected to perform a number of interventions per year in order to maintain proficiency (29). Higher operator volumes are associated with lower in-hospital mortality (30) and adequate reporting of operators’ experience and previous training is important for translation of evidence to different clinical settings (31).

Item 8 (When and how much) was not reported in the majority of trials, both in the registry or the publications. Shortness of intervention in invasive cardiology is associated with the success rate of the intervention and prolonging the intervention is inversely related to the time that has elapsed since its beginning (32) as well as less periprocedural complications (32, 33).

Items 9 (Tailoring), 10 (Modifications), 11 (Planned adherence to the intervention procedure) and 12 (Actual adherence to the intervention procedure) were considered noncompliant if not reported or not clearly considered unnecessary in the study. This might overestimate the level of incompleteness in both registries and publications, but we consider these items of utmost importance for trials in invasive cardiology, where procedures are often modified according to the individual patients.

Item 9 (Tailoring) was not reported for most of analyzed trials, both in the registry and matching publications, even though it is periprocedurally done in everyday clinical practice, for example by choosing the type and dimensions of artificial heart valve (34) or coronary stent size, which directly impacts success rate and number of adverse events (35). Not reporting preparatory steps for the intervention, which enable tailoring to individual patients, impedes the appropriate application of findings.

Modifications during the course of study (Item 10) were not recorded for the majority of trials in both the registry and in the published articles. Reporting of modification of procedures in non-pharmacological research is vital in clinical research to foster safe and efficient innovation, as new procedures and devices undergo a series of improvements during the development before entering clinical practice (36) and clinical trial registries allow providing additional and updated information.

Despite careful planning, changes in interventions are sometimes necessary. Public health crises, such as the COVID-19 pandemic or the Russian invasion of Ukraine led to changes in delivery of interventions (37, 38) by including in-home study visits, distribution of experimental drugs to participants’ homes or implementing other remote monitoring initiatives (39). Our initial search was conducted before the onset of these crises which could explain, to a degree, poor reporting of modifications during studies.

Adherence to interventions, either planned or actual, was not reported for the majority of trials. Adherence is usually linked to pharmacological trials and different approaches to its measurement have been developed (40). Measuring adherence in non-pharmacological interventions is more complex (41), and adherence models have been developed for educational and behavioral interventions (42). Adherence in non-pharmacological, manual studies, such as surgery and invasive cardiology is evaluated through implementation of operative and procedural checklists (43, 44). Ensuring the use of procedural checklists and reporting could reduce postinterventional complications (45) and perhaps enable better adoption of novel interventions in different clinical settings.

More than 80% of trials in our study were industry sponsored. Clinical trials in invasive cardiology have substantial industry involvement (46). Industry-sponsored research is more likely to be published (47) and report favorable study results (48) and these differences cannot be explained by standard risk of bias assessment alone (49). Low adherence to the TIDieR checklist in industry-funded research could be explained by patent policy, especially in the *Clinicaltrials.gov* registry. While data published in journals remains the most important for informing clinical practitioners, trial registries are an important source of information as well as they are often the only available source of information for unpublished research (27).

Publish or perish in a known dilemma in medical research and publication of scientific papers before patents can lead to patent rejection (50); patentable inventions can often be published fully after the delay necessary to legally protect intellectual ownership. In our study, more TIDieR items were reported in the published corresponding articles than in *Clinicaltrials.gov* – a finding similar to the other study involving periodontology intervention trials (27), demonstrating that reporting of interventions improves in publications, but is still below the desired level of completeness and transparency. Updating the entries in clinical trial registries is possible and should be done once the patent application process is complete. Even though the publication of scientific research can be delayed, it should not be postponed indefinitely to protect patentable results (51) and our results show that this is still an issue, even after the publication in the scientific journals.

TIDieR checklist is an important tool to ensure rigorous reporting of non-pharmacological interventions. Trial registries serve as a key regulatory tool, and evidence shows that results are often withheld or incompletely reported. Both Food and Drugs Agency and European Medicines Agency have increased their effort to ensure reporting of results within a year of trial completion (52). In the future, adopted TIDieR checklist could be implemented in the clinical trial registry submission forms, to ensure adequate and complete reporting of results.

### 4.1. Strengths and limitations

This is the first study, to the best of our knowledge, to assess the completeness of reporting of interventions in invasive cardiology. We used a methodologically robust and well-tested TIDieR checklist (6). The strength of our study is also reflected in independent assessments and extraction of the registries and matching publications. The limitation of our study may be that we did not identify all relevant trials. Our search strategy was based around the term ‘invasive cardiology’, and possibly omitted registered trials that did not provide such wording in the title or the registry text. Our strategy, however, retrieved a whole spectrum of invasive cardiology trials (e.g., valvular disease, coronary disease, pacemakers, and electrophysiology studies). We searched a single clinical trial registry and included registry items with results only, thus narrowing our sample size. *Clinicaltrials.gov* is the largest public clinical trial database with more than 460 thousand registrations (53). Additionally, while *Clinicaltrials.gov* registry should provide all necessary information regarding the study, the registry cannot be used as a substitute for a protocol and more information were potentially available in the study protocols, which were not analyzed in this study. Finally, although we used a sensitive search strategy and several databases to retrieve all published articles, there is a possibility that we did not identify all available publications.

## 5. Conclusion

Reporting of interventions in invasive cardiology registered in *Clinicaltrials.gov* and published in journal articles is low. Endorsement and full implementation of the TIDieR checklist in registration and journal submission policies and procedure and thorough regulatory reforms is necessary to improve the reporting of interventions and thus advance evidence-based patient care.

## Data availability statement

The datasets presented in this study can be found in online repositories. The names of the repository/repositories and accession number(s) can be found at: https://osf.io/bx7sc/.

## Ethics statement

We analyzed the data publicly registered in *ClinicalTrials.gov* and published in publicly available scientific journals and an ethics approval is not required.

## Author contributions

VL: Conceptualization, Data curation, Formal analysis, Methodology, Writing – original draft, Investigation. HA: Data curation, Investigation, Methodology, Writing – review & editing. MV: Conceptualization, Methodology, Supervision, Writing – review & editing, Data curation, Formal analysis, Investigation, Writing – original draft. AM: Data curation, Investigation, Methodology, Writing – review & editing, Conceptualization, Funding acquisition, Resources, Supervision, Formal analysis.

## Footnotes

^1^ https://classic.clinicaltrials.gov/

^2^https://www.medcalc.org; 2016.
