# Supplementary material for: Completeness of intervention description in invasive cardiology trials: an observational study of ClinicalTrials.gov registry and corresponding publications
Source: Front Med (Lausanne). 2023 Oct 10;10:1276847. doi: 10.3389/fmed.2023.1276847 (PMC10597631; doi:10.3389/fmed.2023.1276847)
Supplement: Supplementary file 1 [file Table_1.docx]

| **Condition** | **Number (%)** |
| --- | --- |
| **Arrythmias and conduction disorders:** | 120 (39.8) |
| Arrythmia ablation procedures, delivery systems and wires | 59 (19.6) |
| Pacemakers and Implantable cardiac defibrillators | 22 (7.3) |
| Other | 39 (12.9) |
| **Coronary artery disease:** | 99 (32.9) |
| Stents | 44 (14.6) |
| Catheters, wires, and delivery systems | 16 (5.3) |
| Other | 39 (12.9) |
| **Heart failure:** | 43 (14.3) |
| Cardiac resynchronization therapy | 13 (4.3) |
| Ventricular assist devices | 9 (3.0) |
| Other | 21 (7.0) |
| **Valvular disease:** | 23 (7.6) |
| Transcatheter valves | 21 (7.0) |
| Other | 2 (0.7) |
| **Congenital heart defects:** | 7 (2.3) |
| Occluders | 7 (2.3) |
| **Other^1^** | 9 (3.0) |

**Supplement table 1.** Most common interventions registered sorted according to the research category (n=301)

1. Includes arterial hypertension (1 trial), pulmonary embolism (4 trials), ultrasound assistance (2 trials) and invasive heart monitoring for invasive procedures and transitory ishemic attacks (2 trials)
